# Biological learning curves outperform existing ones in artificial intelligence algorithms

**DOI:** 10.1038/s41598-019-48016-4

**Published:** 2019-08-09

**Authors:** Herut Uzan, Shira Sardi, Amir Goldental, Roni Vardi, Ido Kanter

**Affiliations:** 1 Department of Physics, Bar-Ilan University, Ramat-Gan, 52900 Israel; 2 Gonda Interdisciplinary Brain Research Center and the Goodman Faculty of Life Sciences, Bar-Ilan University, Ramat-Gan, 52900 Israel

**Keywords:** Computer science, Learning algorithms

## Abstract

Recently, deep learning algorithms have outperformed human experts in various tasks across several domains; however, their characteristics are distant from current knowledge of neuroscience. The simulation results of biological learning algorithms presented herein outperform state-of-the-art optimal learning curves in supervised learning of feedforward networks. The biological learning algorithms comprise asynchronous input signals with decaying input summation, weights adaptation, and multiple outputs for an input signal. In particular, the generalization error for such biological perceptrons decreases rapidly with increasing number of examples, and it is independent of the size of the input. This is achieved using either synaptic learning, or solely through dendritic adaptation with a mechanism of swinging between reflecting boundaries, without learning steps. The proposed biological learning algorithms outperform the optimal scaling of the learning curve in a traditional perceptron. It also results in a considerable robustness to disparity between weights of two networks with very similar outputs in biological supervised learning scenarios. The simulation results indicate the potency of neurobiological mechanisms and open opportunities for developing a superior class of deep learning algorithms.

## Introduction

**Figure 1:**
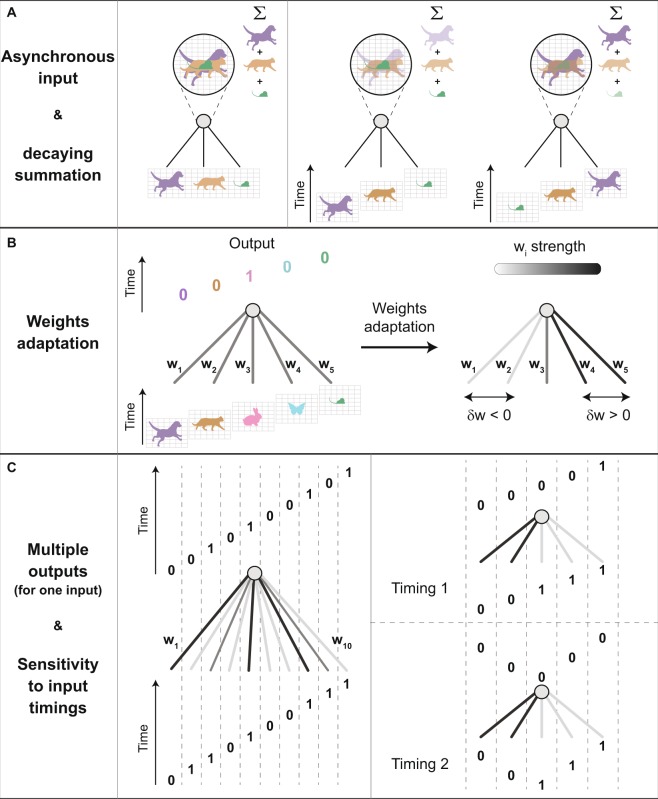
Schematic of the main three dynamical features that differentiate biological and traditional perceptrons. (**A**) Synchronous versus asynchronous inputs. Left: Traditional synchronous perceptron with three equal weights, where its output linearly sums up the inputs. Right: Perceptrons with asynchronous inputs, and their decaying summation by the output unit. Different temporal orders of the inputs result in different outputs. (**B**) An adaptation scheme similar to spike-timing-dependent-plasticity (STDP). Left: A perceptron with five asynchronous inputs and five outputs, respectively. A sub-threshold stimulation and a spike are denoted by 0 and 1, respectively. Right: Preceding stimulated weights to the evoked spike, w_1_ and w_2_, are weakened, whereas the latter ones, w_4_ and w_5_, are strengthened (strengths are gray-coded). (**C**) Multiple outputs for an asynchronous input. Left: A perceptron with ten weights (strengths are gray-coded) and an asynchronous input with multiple evoked spikes. Right: Two different timings of the same asynchronous input result in different asynchronous outputs.

A primary objective of artificial learning algorithms is to pinpoint and classify the many objects that compose an event based on their relative timings. A commonly used strategy is to reduce the complexity of such an event to synchronous inputs, and analyze it using feedforward networks^1–6^ (Fig. 1A, left). In literature, this strategy has been extensively evaluated using rule-based statistical physics and non-linear dynamics methods^7–20^.

Unlike modern computers^21^, the dynamics of the brain do not comply with a well-defined global clock^22–24^, which is a characteristic of physical systems that obey random sequential updating. Hence, the biological scheme must cope with asynchronous inputs^21,25^ (Fig. 1A, right). This objective is achieved using decaying input summation and the spike-timing-dependent-plasticity (STDP)^26,27^ mechanism. In STDP, a weight that induces a sub-threshold signal on a neuron is weakened or strengthened based on its relative timing to adjacent spikes, generated by the same neuron (Fig. 1B). However, the weights that induce above-threshold signals, i.e. spikes, do not undergo adaptation. Moreover, the biological asynchronous scheme allows multiple outputs for a given input (Fig. 1C, left), as well as different outputs for different timings of the same asynchronous input (Fig. 1C, right). These features (Fig. 1), for instance, are unique to the biological scheme and cannot be realized by its synchronous version.

Although asynchronous inputs contain more information than their synchronous counterparts, they require a practical mechanism that can utilize the timing information contained in the input units (Fig. 1). While biological adaptation considers asynchronous inputs, its quantitative effect on the learning rate is still unclear. Hence, it is fair to conclude that the current concept of biological adaptation stemming from asynchronous inputs^26,28^ has been developed with limited influence^25,29–31^ on the current advanced deep learning algorithms^1–6^.

In essence, the brain is a complex network containing billions of nodes (neurons), where each node communicates simultaneously with thousands of other nodes via their links (synapses). However, each node integrates its incoming signals through several long ramified “terminals”, dendritic trees. Recently, a new type of a cooperative nonlinear dynamics was proposed, wherein the adaptation is attributed solely to the several nodal terminals^32^, instead of the network links. This dendritic adaptation presents a self-controlled mechanism to prevent divergence or vanishing of the adaptive parameters in the biological scheme, as opposed to the trend of synaptic plasticity, adaptive links. In addition, it supports self-oscillations of the effective adaptive parameters^32^. However, the learning capabilities of such cooperative nonlinear dynamics are not fully known; this is in contrast to the extensive literature available on the learning capabilities of existing deep learning algorithms^1–6^.

This work sets out to demonstrate that, compared with the existing artificial intelligence algorithms, asynchronous input based biological learning schemes can improve the scaling of learning rates of feedforward networks. We also show that the generalization error in supervised learning of multi-layer feedforward networks is independent of the size of the input, and rapidly decreases with the number of examples.

First, the biological adaptation mechanism for asynchronous inputs, which is similar to STDP, and the phenomenon of decaying input summation are described. Then, using large-scale simulations, the generalization error for such biological perceptrons under synaptic plasticity is estimated. The simulation results indicate fast learning rates where the generalization error rapidly decreases with increasing number of examples, independent of the size of the input.

Next, we explore dendritic adaptation, which is based on a new mechanism of swinging between reflecting boundaries of the time-dependent dendritic strengths. The results suggest that faster learning rates can be achieved with dendritic adaptation in comparison to a traditional synchronous perceptron. In addition, an extension of the fast learning rates to multi-layer networks is also discussed. The paper concludes with guidelines for fundamental questions in the future regarding the development of advanced classes of deep learning algorithms.

## Results

**Figure 2:**
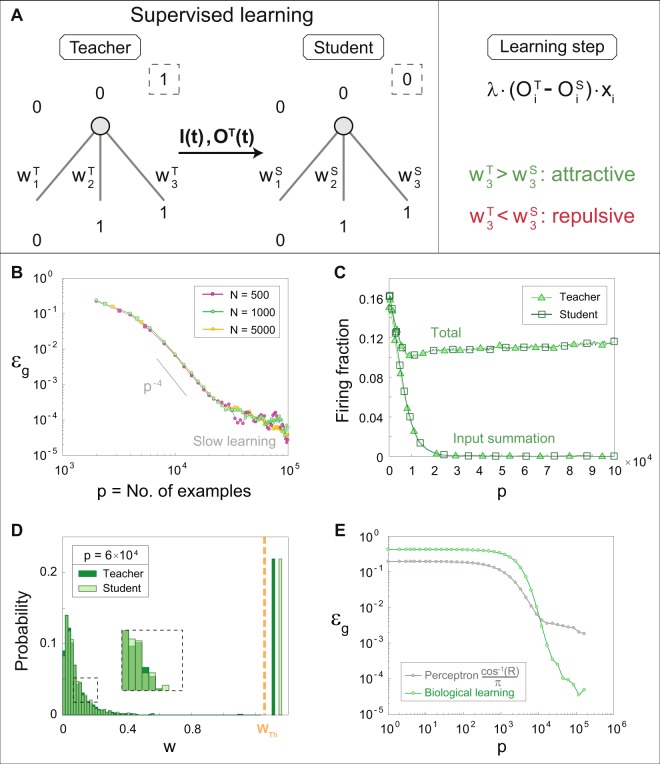
Supervised learning rates using synaptic learning. (**A**) A scheme of supervised learning (left) indicating that the information provided for the student is the asynchronous input example, I(t), and the output timings of the evoked spikes of the teacher, O^T^(t). A learning step (right) occurs for weights of the student with conflicting outputs, Eq. (2) (w_3_^S^ in the example on right). This learning strategy implements either attractive or repulsive steps, but the majority are attractive (Fig. S8). (**B**) The generalization error, ε_g_, scales with the number of continuous input examples, p, (Methods) which initially decreases as p^−ϕ^, ϕ > 1. Power-law slope of p is presented as a guideline. (**C**) The fraction of fires for each example in (**B**) for Ν = 1000 and the fraction of fires resulted from temporal input summation only. (**D**) The probability distribution, including a zoom-in, of the weights of the teacher (dark green) and the student (light green) for (**B**) with Ν = 1000 and at p = 6 · 10^4^. The weight threshold, w_Th_, is set to 1.25 as the minimal continuous input is 0.8 (Methods), and the above-threshold strengths are depicted arbitrarily. (**E**) ε_g_ for (**B**) with Ν = 1000; comparison with ε_g_ based on the normalized overlap between the weights of the teacher and the student, R (Methods). Error bars in panels (B,E) are less than twice the size of the symbols.

Let us consider a scenario where a biological perceptron performs supervised learning^17,33^ (Fig. 2A). In general terms, the objective of a student is to imitate the response of the teacher, where both the student and the teacher have the same architecture^34^, comprising Ν asynchronous input units, and are characterized by the dynamics of synaptic plasticity (Fig. 1B). The teacher is initially defined by a set of Ν weights, $\{{w}_{m}\}$, where its leaky integrate-and-fire (LIF) output neuron^35^ is updated following a serial representation of p examples, i.e., asynchronous inputs (Methods). Thus, each example is said to contain Ν randomly ordered presynaptic spikes, one for each input unit (Fig. 1C, left) resulting in decaying summation by the output unit (Methods), which produces a set of spikes timings for each example. Next, synaptic adaptation is implemented using a version of STDP, for each stimulated weight, which does not result in an evoked spike

1 $${w}_{m}^{+}={w}_{m}\cdot (1\pm \delta (t))$$

Here, ${w}_{m}^{+}$ indicates the updated m^th^ weight and $\delta (t)$ indicates the strengthening/weakening of a weight conditioned to a prior/later evoked spike at a time delay $t$ in a given time window (Methods and Fig. S1).

The information provided to the student for each example is the input and the timings of evoked spikes of the teacher (Fig. 2A). For each example, the dynamics of the student consist of the following three steps. First, the outputs are produced and its weights are adapted according to the STDP of the teacher. Note that very similar qualitative results were obtained where the student performs the STDP following the student’s outputs instead of the teacher’s outputs (Fig. S2). Then, learning is performed, wherein the weights with conflicting outputs between the teacher and the student are modified towards an expected agreement (Fig. 2A, right). This learning rule is similar to the traditional synchronous perceptron learning step and can be seen as minimization of a cost function (Methods)

2 $${w}_{m}^{+}={w}_{m}+\lambda \cdot ({O}_{m}^{T}-{O}_{m}^{S})\cdot {x}_{m}$$

where ${O}_{m}^{T}$ and ${O}_{m}^{S}$ indicate the output of the teacher and the student at the time of the m^th^ input, respectively; ${\rm{\lambda }}$ denotes the learning step size, and ${x}_{m}$ stands for the input amplitude of ${w}_{m}$. The simulation results for various input sizes, Ν, indicate that the generalization error, ε_g_, scales with p, independent of Ν (Fig. 2B). This extremely fast scaling for the learning rates is compared with the traditional, much slower, optimal learning rate^17,18^, ε_g_ ∝ 1/α, for the synchronous perceptron, where α = p/Ν. Its underlying dynamical mechanism is suggested by a crossover from ε_g_ ∝ p^−ϕ^, ϕ > 1, to a slow learning phase where ϕ $\approx$ 1 (Fig. 2B). In the first phase, the sub-threshold weights dominate the dynamics, and most of the output spikes are due to the temporal summation of several temporally consecutive sub-threshold inputs (Fig. 2C). Consequently, a fraction of above-threshold weights was found to be identical for both the teacher and the student (Fig. 2D), which from now on their evoked spikes do not contribute to the learning process, as suggested by Eq. (2). Thus, the learning process is now dominated by low probability events of conflicting outputs, emerging from temporal summation of stimulations of weak weights (Fig. 2D). The probability of finding several weak stimulations consecutively is low; moreover, the weak weights are very similar for both the teacher and the student (Fig. 2D), which significantly reduces the learning rate (Fig. 2B).

In addition to accelerated learning rates, which scale with p, biological learning also exhibits robustness of ε_g_ for a dissimilarity between the weights of the student and the teacher in the late stage of the dynamics (Fig. 2E). For the synchronous perceptron scheme, ε_g_ is a function of the normalized overlap, R (Methods), between the weights of the teacher and the student^17^ (gray line in Fig. 2E), which is eliminated only when the weights are identical, i.e., R = 1. However, for the biological scheme, the dynamical effect of above-threshold weights is independent of their exact values. In addition, the resetting of the membrane potential after their spikes (Methods) reduces the probability for temporal summation of several weak stimulations. Note that the temporal firing of the biological perceptron is very sensitive to the decaying summation of the membrane potential as well as its reset after firing. Those features result in enhanced ε_g_ of the biological learning at the initial learning stage, small p, in comparison to the synchronous perceptron.

**Figure 3:**
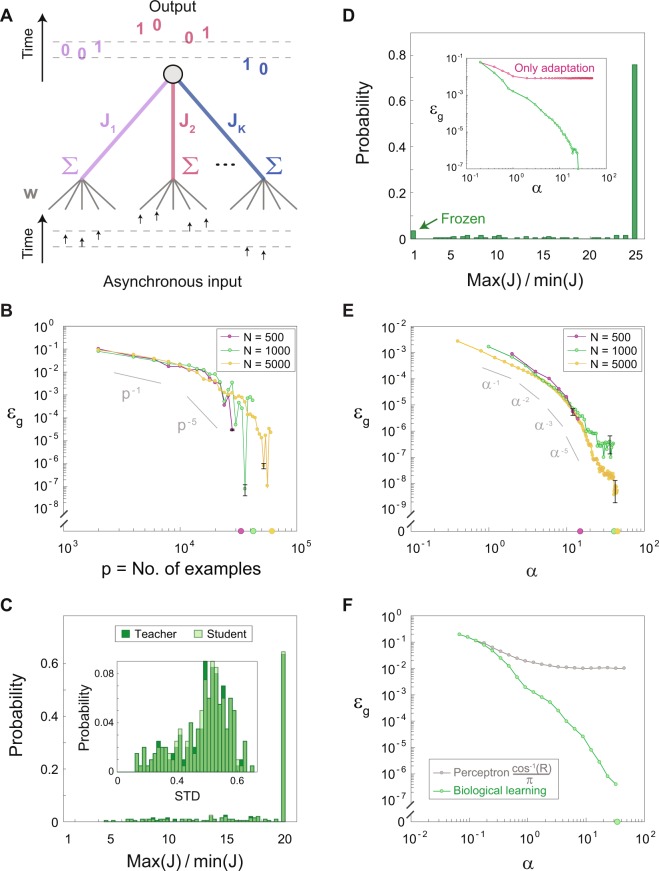
Supervised learning rates using dendritic learning. (**A**) A scheme of an output unit connected to Ν input units via Κ = Ν/5 adaptive dendritic strengths, ${J}_{i}$, where each dendrite connects to five asynchronously stimulated input units via time-independent weights, ${w}_{m}$. The continuous valued inputs of each dendrite are presented asynchronously with a random order that produce multiple output evoked spikes. (**B**) The scaling of the generalization error, ε_g_, with p obtained in simulations using supervised dendritic adaptation only, where finally ε_g_ jumps to zero (Methods). Power-law slopes of p are presented as guidelines. (**C**) The probability distribution of the maximum value of ${J}_{i}$ divided by its minimum value for the student in (**B**) with Ν = 1000 and p > 10^4^. Inset: Probability distribution of the standard deviation of ${J}_{i}$, teacher (dark green) and the student (light green). (**D**) Similar to (**C**), where the dynamics of the student follows the adaptation of the teacher with additional learning step sizes ∝ 1/Ν to ensure the learning of the strengths of a small fraction of J, which freezes in the dynamics. Inset: ε_g_ with adaptation following the teacher only (pink) and with additional learning (green) (Methods). (**E**) The scaling of ε_g_, with α obtained in simulations as for (**D**). Power-law slopes of α are presented as guidelines. (**F**) ε_g_ from (**E**) for Ν = 1000 (green); comparison of ε_g_ obtained from the normalized overlap, R, between the dendritic strengths of the teacher and the student (gray). Error bars for panels (B), (E), (F) and ε_g_ > 5 ∙ 10^−5^ are less than twice the size of the symbols; only the maximum for each panel is presented (Methods).

Recently, several experimental and theoretical studies have suggested that dendritic adaptation is a paradigm shift in brain learning^32^, as opposed to learning based solely on slow synaptic plasticity. The adaptation process in the form of STDP is attributed to ${J}_{i}$, which indicates the strengths of the terminals (dendrites) of the neuron^36^; the weights, ${w}_{m}$, are time-independent (Fig. 3A and Methods). This is representative of non-local adaptation, where many incoming weights, ${w}_{m}$, to the same terminal (dendrite) concurrently undergo the same adaptation. In the new paradigm, the network dynamics is now counterintuitively governed by the weak ${w}_{m}$, which were previously assumed insignificant. Thus, a novel self-controlled mechanism is presented to prevent divergence or vanishing of the learning parameters, as opposed to synaptic adaptation (Figs 2D and S3); the new paradigm also supports self-oscillations of the adaptive parameters, ${J}_{i}$ (Fig. S4).

The supervised learning process of the student under dendritic adaptation, following the teacher and without learning steps, indicates that ε_g_ decays with p, and is independent of the size of the input, Ν (Fig. 3B). In addition, after a considerable decay, ε_g_ jumps to zero at p, which slightly increases with Ν (Fig. 3B). This type of learning with adaptation is a consequence of the temporal oscillating nature of ${J}_{i}$ (Figs 3C and S4); conversely, ${w}_{m}$ converge to extreme values under synaptic plasticity (Figs 2D and S3). Hits at the boundary values form the underlying mechanism for tracking between the strengths of the oscillating ${J}_{i}$ of the teacher and the student (Fig. S5 and Methods). Each hit of a given ${J}_{i}$ decreases its gap between the teacher and the student, because both increase or decrease their strengths simultaneously^37^. The synchronization of the last ${J}_{i}$ eliminates ε_g_, which occurs at p that slightly increases with Ν, as it takes more time to synchronize more random walkers (although not independently) with reflecting boundaries^38^.

For the case of dendritic adaptation, it is likely that during the dynamics a small fraction of the dendritic strengths will become dynamically frozen (Fig. 3D,E). A frozen dendritic strength emerges when all its effective weights, ${w}_{m}\cdot {J}_{i}$, are above-threshold, i.e. evoke spike for every stimulation; hence its adaptation is terminated. Therefore, the tracking of these frozen dendritic strengths by the student is terminated, those events are reflected by a plateau in ε_g_; however, the addition of learning steps for ${J}_{i}$ (similar to Eq. (2)) results in a fast decrease of ε_g_ (Fig. 3D, inset). For a learning step size ∝ 1/Ν, ε_g_ scales with α^−ϕ^; ϕ can increase beyond 1 (Fig. 3D,E), which is defined as scaling for the optimal learning rate for the synchronous perceptron. For a fixed learning step size, independent of the size of the input, ε_g_ scales with p (Fig. S6), similar to the initial scaling of synaptic learning rates (Fig. 2B), however, with a final jump to ε_g_ = 0. The learning process enables the student to track the strengths of the frozen ${J}_{i}$; hence, it scales with α for Ο(1/Ν) learning step size and with p for finite step size.

Dendritic learning also exemplifies the robustness of ε_g_ to a dissimilarity between the weights of the teacher and the student along the dynamics (Fig. 3F). In the initial adaptation process (α $\lesssim$ 0.2 in Fig. 3F), oscillating ${J}_{i}$ are mainly synchronized by hits at their boundary values, and ε_g_ is mainly a function of the normalized overlap, R, between the weights of the teacher and the student. In the rest of the dynamics, the small fraction of frozen ${J}_{i}$ is learned by the student until ε_g_ is eliminated; however, their exact above-threshold values are irrelevant (Fig. S7).

**Figure 4:**
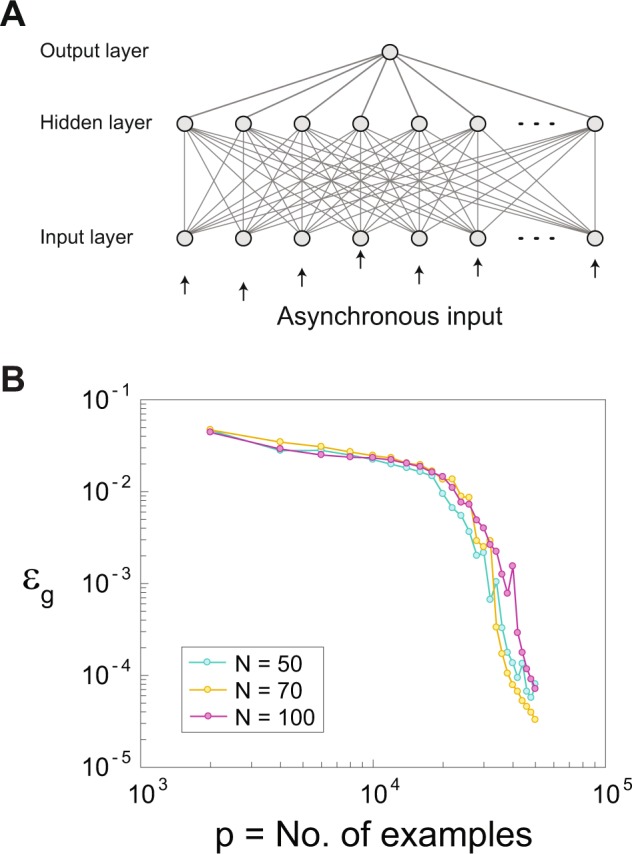
Supervised learning rates for a multilayer network. (**A**) A scheme of a fully connected multilayer network with one hidden layer. The number of input units is N as well as the number of hidden units. The input layer units are asynchronously stimulated (Methods). (**B**) The generalization error, ε_g_, scales with the number of examples, p, similar to Fig. 2B, for the dynamics of synaptic learning (Methods).

Preliminary results indicate that similar qualitative trends are also valid for other multilayer networks with one hidden layer in the synaptic scenario. In particular, the network consists of an input layer which was fully connected to a hidden layer; each layer comprised N units, and a single output unit (Fig. 4). All hidden units and the output unit functioned as LIF neurons (Methods). The results suggest that the generalization error, ε_g_, scales with the number of examples, and it is independent of the size of the input. In addition, preliminary results indicate that the qualitative conclusions reported here for the dendritic scenario are applicable also for multilayer networks, which, however, deserve further research.

## Discussion

In this work, we demonstrate that learning rates of state-of-the-art artificial learning algorithms can be improved by adopting fundamental principles that govern the dynamics of the brain. The theory of supervised learning in feedforward networks trained by random examples, suggests that the optimal generalization error scales as $1/\alpha \,$, where $\alpha$ is the ratio between the number of examples and the number of input units. This scaling is valid for on-line as well as for off-line learning and is based on synchronous inputs. In other words, all the input units of the feedforward network are stimulated simultaneously, and all delays are equal. The brain essentially analyses complex data, which is abundant in life, using very slow and noisy asynchronous dynamics^39^. The objective of supervised learning in such an environment appears much more complex and difficult when compared with the synchronous version, even though these asynchronous inputs contain more information about the relative timings of stimulations arriving from different input units. Despite the apparent complexity, the demonstrated biological learning rates outperform the existing artificial ones, where the generalization error decays with the number of examples, and it is independent of the number of input units. For adaptive weights (synaptic plasticity), in addition to biological adaptation (STDP), these fast learning rates can be achieved using learning steps. However, for the new type of cooperative dynamics, dendritic adaptation, the fast decay of the generalization error is achieved only by using adaptation, without the learning steps. The new type of cooperative dynamics is based on a new mechanism of swinging between reflecting boundaries for the time-dependent dendritic strengths. Herein, hits at the boundaries result in attractive steps between the teacher and the student networks and provide an example for adaptation as a learning mechanism.

The realization of asynchronous inputs in artificial systems using the synaptic scenario is on the one hand more complex, since the dynamics consist of adaptation and learning, whereas in synchronous inputs scenario only learning steps take place. On the other hand, a learning step for synchronous input scenario requires the update of all weights, whereas for the asynchronous case only a weight with mismatch output is updated, and in addition the adaptation is implemented only for neighboring weights in a defined time window. A similar tradeoff holds for the implementation of supervised learning in multilayer networks and the question which method, synchronous or asynchronous inputs, is more time-consuming depends on the detailed architecture and dynamical rules.

For the dendritic scenario, the number of tunable parameters is much smaller compared to the synaptic scenario. For instance, the number of tunable parameters between the input and the hidden unit in a multilayer network with one hidden layer (Fig. 4) is N^2^ for the synaptic case and equals to [number of dendrites * N] for a similar dendritic network, where N is the number of input units and hidden units. Hence, the implementation of learning in the dendritic scenario is characterized by a reduced time-consumption in comparison to its similar synchronous inputs version as well as to its asynchronous synaptic version.

The framework of dendritic adaptation results in time-dependent dendritic strengths even for the teacher network; hence, a change in the working premise - from a static teacher to a dynamic teacher - is required. The time separation between training and generalization procedures, which is simple to utilize in the analysis of supervised learning of artificial networks, is invalid in the biological scenario. The theoretical analysis of such a biological reality requires the development of new methods and tools. In addition, the reproducibility of the activity of such networks, i.e. input-output relations, on long time scales has to be examined. Given that the network adaptive parameters are time-dependent, the same input is expected to produce different outputs for significant separated timings. This may be attributed to the large number of intermediate inputs and adaptation steps. Therefore, such a scenario might require reconsideration of the basic definitions of quantities such as capacity and learning a rule in supervised and unsupervised learning. The development of artificial learning towards these new goals and concepts might be achieved in the foreseeable future by perturbation around the current static teacher assumptions. It is also intriguing to investigate whether other types of artificial adaptive teachers, beside the biological prescription, also enhance learning.

The generalizations of the presented results to recurrent networks^40–42^ is intriguing and especially the estimation of the scaling of quantities such as number of attractors, their basin of attractions and capacity. In addition, it is intriguing to generalize the proposed biological model to Hamiltonian spin systems^43^ or to binary neuronal models, similar to the existing ones in computer science and statistical mechanics. This might lead to analytical solutions using the existing methods developed for such systems, as well as simplified simulations, which will shed light on learning in the general class of such artificial systems.

## Methods

### The feedforward network

The network consists of an input layer with N input units and an output unit functioning as a leaky integrated and fire (LIF) neuron (see Output production). The input units are connected to the output unit via N synaptic weights, ${w}_{m}$ (Fig. 2), or via K = N/5 dendritic strengths, ${J}_{i}$ (Fig. 3). In the synaptic scenario, $\{{w}_{m}\}$ are the tunable parameters (Fig. 2), whereas for the dendritic scenario, $\{{J}_{i}\}$ are the tunable parameters while $\{{w}_{m}\}$ are time-independent (Fig. 3).

### The supervised learning algorithm

The scenario of supervised learning by a biological perceptron is examined using a teacher and a student. The mission of the student is to imitate the responses, i.e. the outputs, of the teacher, where both have the same architecture. For each input the teacher produces an output. The timings and the amplitudes as well as the resulting teacher’s outputs for each input unit, are provided to the student. Those input/output relations constitute the entire information provided to the student for each input. The algorithm is composed of 3 parts: output production, weights adaptation and learning.

Output production: An identical asynchronous input, example, is given to the teacher and the student, each produces its output according to their weights and decaying input summation, O^T^ and O^S^, respectively (see Output production – Leaky integrate and fire neuron).

Weight adaptation: For each input unit the teacher preforms weights adaptation next to its output production, following its input/output (see Adaptation). The student preforms the same adaptation as the teacher, unless otherwise stated (see Student’s adaptation).

Learning: The student preforms learning steps, unless otherwise stated, on weights with conflicting outputs with the teacher, i.e. O_m_^T^ ≠ O_m_^S^ for the m^th^ input unit.

### Inputs generation

Each input is composed of N/2 randomly stimulated input units. For each stimulated unit a random delay and a stimulation amplitude are chosen from given distributions. The delays are randomly chosen from a uniform distribution with a resolution of 0.01 ms, such that the average time-lag between two consecutive stimulations is 5 ms. Stimulation amplitudes were randomly chosen from a uniform distribution in the range [0.8,1.2]. Note that the reported results are qualitatively robust to the scenario where all non-zero amplitudes equal 1. In the dendritic scenario, the five ${w}_{m}$ connected to the same dendrite were stimulated sequentially in a random order and with an average time-lag of 5 ms between consecutive stimulations.

### Output production – Leaky integrate and fire neuron

In the synaptic adaptation scenario, the voltage of the output unit is described by the leaky integrate and fire (LIF) model

3 $$\begin{array}{c}\frac{dV}{dt}=-\,\frac{V-{V}_{st}}{{\rm T}}+\mathop{\sum }\limits_{m=1}^{N}{w}_{m}\delta (t-{\tau }_{m})\end{array}$$

where V(t) is the scaled voltage, Τ = 20 ms is the membrane time constant and V_st_ = 0 stands for the scaled stable (resting) membrane potential. ${w}_{m}$ and τ_m_ stand for the m^th^ weight and delay, respectively. A spike occurs when the voltage crosses the threshold, V(t) ≥ 1 and at that time the output unit produces an output of 1, otherwise the output is 0. After a spike occurs, the voltage is set to V_st_. For simplicity, we scale the equation such that V_th_ = 1, V_st_ = 0, consequently, V ≥ 1 is above threshold and V < 1 is below threshold. Nevertheless, results remain the same for both the scaled and unscaled equations, e.g. V_st_ = −70 mV and V_th_ = −54 mV. The initial voltage was set to V_(t = 0)_ = 0.

In the dendritic adaptation scenario, the voltage of each dendritic terminal is described by

4 $$\begin{array}{c}\frac{d{V}_{i}}{dt}=-\,\frac{{V}_{i}-{V}_{st}}{{\rm T}}+{J}_{i}\cdot \mathop{\sum }\limits_{m=\frac{N}{K}(i-1)+1}^{\frac{N}{K}\cdot i}{w}_{m}\delta (t-{\tau }_{m})\end{array}$$

where V_i_(t) and ${J}_{i}$ stand for the voltage and the strength of the i^th^ dendrite, respectively. The rest of the parameters are identical to the synaptic adaptation scenario.

### Adaptation

The adaptation for the synaptic scenario, is done according to

5 $$\begin{array}{c}{w}_{m}^{+}={w}_{m}\cdot (1+\delta (t))\end{array}$$

where t is the time-lag between a sub-threshold stimulation to ${w}_{m}$ (stimulation that didn’t evoke spike, output 0) and an evoked spike. Similarly, the dendritic adaptation is given by

6 $$\begin{array}{c}{J}_{i}^{+}={J}_{i}\cdot (1+\delta (t))\end{array}$$

where t now is the time-lag between a sub-threshold stimulation at ${J}_{i}$ and an evoked spike from another dendrite. For both scenarios

7 $$\begin{array}{c}\delta (t)=A\cdot \exp (-\frac{t}{15})\cdot sign(t)\end{array}$$

representing the strengthening/weakening of a weight conditioned to a prior/later evoked spike at a time delay t, respectively, where a cutoff time window of 50 ms is enforced (Fig. S1). For simplicity, a step function,

8 $$\begin{array}{c}\delta (t)=\pm \,A\end{array}$$

was used for all time delay t, unless otherwise stated. However, all results are robust to adaptation in the form of either exponential decay or a step function.

### Student’s adaptation

In order to perform the same adaptation as the teacher, the required information is the teacher’s temporal input/output relations. Note that although the student performs the same adaptation steps as the teacher, it does not necessarily ensure tracking of the parameters of the teacher, since the changes in the weights are relative to the current value of the weights of the student.

### Learning

Learning steps were performed on the student’s weights with conflicting output with the teacher. This learning rule is based on a gradient descent dynamics, which minimizes a cost function

$${C}_{m}=({V}^{T}-{V}^{S})\cdot ({O}_{m}^{T}-{O}_{m}^{S})$$

that measures the deviation of the student voltage form the teacher voltage in case of an error (unmatched spike timings between the teacher and the student). A spike is considered as V=1. The change in the weights ${w}_{m}$ is proportional to the negative derivative of the cost function relative to the weight.

$$\begin{array}{ccc}{\rm{\Delta }}{w}_{m}\propto -\,\frac{d{C}_{m}}{d{w}_{m}} & = & -\frac{d{C}_{m}}{d{V}^{S}}\frac{d{V}^{S}}{d{w}_{m}}({O}_{m}^{T}-{O}_{m}^{S})=\frac{d{V}^{S}}{d{w}_{m}}({O}_{m}^{T}-{O}_{m}^{S})\\  & = & {x}_{m}\exp (\frac{t-{\tau }_{m}}{T})({O}_{m}^{T}-{O}_{m}^{S}).\end{array}$$

For simplicity, the weighted exponential prefactor is neglected, but qualitative results remain similar for both cases. Consequently, the learning step for the synaptic scenario is similar to the traditional perceptron learning algorithm

9 $$\begin{array}{c}{w}_{m}^{+}={w}_{m}+\lambda \cdot ({O}_{m}^{T}-{O}_{m}^{S})\cdot {x}_{m}\end{array}$$

and similarly for the dendritic scenario

10 $$\begin{array}{c}{J}_{i}^{+}={J}_{i}+\lambda \cdot ({O}_{m}^{T}-{O}_{m}^{S})\cdot {x}_{m}.\end{array}$$

λ denotes the learning step and O_m_^T^ and O_m_^S^ are the outputs of the teacher and the student at the m^th^ input unit in the i^th^ dendrite, respectively, and ${x}_{m}$ denotes the stimulation amplitude of the m^th^ input unit.

### Calculating the generalization error

The generalization error is estimated every 2000 (1000) inputs in the synaptic (dendritic) scenario. The estimation consists of up to 250,000 inputs presented to the teacher and the student. The generalization error is defined as

11 $$\begin{array}{c}{\varepsilon }_{g}=\frac{total\,no.\,of\,mismatch\,firing\,}{total\,no.\,of\,stimulations}.\end{array}$$

For each measured ε_g_(p) the generalization error is measured at least three times, and the average is presented with the largest error bar among all measured ε_g_(p).

Figure 2: $\{{w}_{m}\}$ were chosen from a uniform distribution in the range [0.1, 0.9] and then were normalized to a mean equals to 0.5. Adaptation was following Eq. (8) with A = 0.003, and learning was following Eq. (9) with λ = 1/1000. ${w}_{m}$ was bounded from above by 1.5 and from below by 10^−4^.

In panel B, the STD of the generalization error was in the order of the size of the circles, and therefore not shown in the graph.

In panel C, two types of firing fractions are presented. The first consists of the total number of spikes normalized by N. The second indicates the number of spikes induced by input summation normalized by N, i.e. evoked spikes by weights obeying ${w}_{m}\cdot {x}_{m} < 1$.

In panel D, the normalized histogram of the synaptic weights at p = 6 * 10^4^ is plotted using 75 bins.

In panel E, the error of a perceptron was calculated according to

12 $$\begin{array}{c}{\varepsilon }_{g}=\frac{1}{\pi }{\cos }^{-1}(R)\end{array}$$

where R is the normalized overlap between the teacher’s and the student’s weights

13 $$R=\frac{({w}^{S}-1)\cdot ({w}^{T}-1)}{||{w}^{S}-1||\cdot ||{w}^{T}-1||}.$$

Figure 3: $\{{w}_{m}\}$ were chosen from a uniform distribution in the range [0.1, 0.9] and then were normalized to a mean equals to 0.5. $\{{J}_{i}\}$ were chosen from a uniform distribution in the range [0.5, 1.5].

In panel B, stimulations with low amplitudes (0.01) were given to the N/2 unstimulated input units, resulting in non-frozen ${J}_{i}$. In addition, adaptation was performed according to the spike timings of the two prior and two later stimulated dendrites, instead of using a cutoff in time. This modification was introduced to overcome the order of the delays in our setup, i.e. delays of w belonging to dendrite i are greater than delays of w belonging to dendrite i − 1. Consequently, using a cutoff might break the symmetry in the adaptation steps of J (number and strength) before and after the dendrite generating a spike. The adaptation follows Eq. (8) with A = 0.003 and the learning follow Eq. (9) with λ = 1/N. ${J}_{i}$ was bounded from below by 0.1 and from above by 2. The first p where ${J}^{T}$ and ${J}^{S}$ were identical is denoted on the x-axis, ε_g_ = 0.

In panel C, the normalized histogram of 75 bins is presented for the dynamics excluding the transient of the first 10,000 inputs. Similarly, the transient was excluded in the normalized histogram presented in the inset.

In panels D, E adaptation follows Eq. (7) with A = 0.05, and learning follows Eq. (9) with λ = 1/N. ${J}_{i}$ was bounded from below by 0.1 and from above by 2.5. The first p where the non-frozen ${J}^{T}$ and ${J}^{S}$ were identical is denoted on the x-axis, ε_g_ = 0.

In panel F, the error of a perceptron was calculated similarly to the synaptic case, Eqs (12) and (13), with ${J}^{T}$ and ${J}^{S}$ instead of ${w}^{T}$ and ${w}^{S}$, respectively.

Figure 4: The network was composed of an input layer which was fully connected to a hidden layer, each consisted of N units, and a single output unit. All hidden units and the output unit functioned as LIF neurons (see Output production). The fixed delays in the first and the second layer were chosen randomly from a uniform distribution in the range [0, 5N/2] with a resolution of 0.001 ms, where the teacher and the student had the same architecture. Initial weights for both layers were chosen from a uniform distribution in the range [0.1,0.9] and were bounded from above by 1.5 and from below by 10^−4^. An asynchronous input was given to N/2 randomly chosen input units. For each stimulated unit a random delay was chosen from a uniform distribution with a resolution of 0.001 ms, such that the average time-lag between two consecutive stimulations was 5 ms. Stimulation amplitudes were set to 1. The delays were chosen such that stimulation routes from an input unit to the output unit were non-degenerated, i.e. no more than one stimulation arrived simultaneously to a unit. In the first step of the dynamics the hidden layer produced its outputs. Second, those outputs were used as the input to the output unit, which then produced its output. Finally, weights adaptation was performed using Eq. (7) with A = 0.001 and learning following Eq. (9) with λ = 0.002. For the first layer of weights the teacher and the student preformed weights adaptation according to their inputs and outputs of the hidden units. For the second layer of weights, the student performed adaptation according to the teacher’s spike timings and its own sub-threshold stimulation timings. Learning steps were performed on the student’s weights of the first and the second layers with conflicting output with the teacher. Learning was based on the distinct stimulation routes, i.e. for each spike in the teacher’s output, the student knew which hidden unit evoked the spike. The error was calculated every 2000 inputs following Eq. (11), using the upper bound of the total number of stimulations, N^2^/2. For N = 100 all the delays were chosen with a resolution of 0.0001 ms.

Figure S2: Parameters are the same as in Fig. 2, with A = 0.001 (Eq. 8) and λ = 50/N (Eq. 9).

Figure S6: Parameters are the same as in Fig. 3E, with λ = 1/1000 (Eq. 10).

Figure S7: Data extracted from Fig. 3E. The normalized overlap of ${J}_{i}$ was divided to the overlap of frozen and non-frozen dendrites. A frozen dendrite was defined such that the variance of its strengths during the last 500 inputs was less than 10^−3^. For each group the normalized overlap was calculated following Eq. (13) with ${J}^{T}$ and ${J}^{S}$ instead of ${w}^{T}$ and ${w}^{S}$, respectively.

Figure S8: For the synaptic scenario, data was extracted from simulation using the same parameters as in Fig. 2. After each input the following term was calculated for each stimulated weight

14 $$\begin{array}{c}({w}^{T}(p-1)-{w}^{S}(p-1))({w}_{after\,learning}^{S}-{w}_{befor\,learning}^{S})\end{array}$$

measuring whether the learning step decreases the gap between w^T^(p − 1) and w^S^(p − 1). In case the term, Eq. (14), was positive (negative) the step was classified as attractive (repulsive). For the dendritic scenario, data was extracted from simulation using the same parameters as in Fig. 3B, and the attractive/repulsive steps were calculated using Eq. (14) with ${J}^{T}$ and ${J}^{S}$ instead of ${w}^{T}$ and ${w}^{S}$, respectively.

## Supplementary information

Supplementary Information

## Data Availability

All data generated or analyzed during this study are included in this published article (and its Supplementary Information (SI) Files).

## References

[1] Webb S (2018). Deep learning for biology. Nature.

[2] Butler KT, Davies DW, Cartwright H, Isayev O, Walsh A (2018). Machine learning for molecular and materials science. Nature.

[3] Angermueller C, Pärnamaa T, Parts L, Stegle O (2016). Deep learning for computational biology. Molecular systems biology.

[4] Schmidhuber J (2015). Deep learning in neural networks: An overview. Neural networks.

[5] Mnih V (2015). Human-level control through deep reinforcement learning. Nature.

[6] LeCun Y, Bengio Y, Hinton G (2015). Deep learning. nature.

[7] Tramel EW, Gabrié M, Manoel A, Caltagirone F, Krzakala F (2018). Deterministic and Generalized Framework for Unsupervised Learning with Restricted Boltzmann Machines. Physical Review X.

[8] Li B, Saad D (2018). Exploring the function space of deep-learning machines. Physical review letters.

[9] Fösel T, Tighineanu P, Weiss T, Marquardt F (2018). Reinforcement learning with neural networks for quantum feedback. Physical Review X.

[10] Breuer D, Timme M, Memmesheimer R-M (2014). Statistical physics of neural systems with nonadditive dendritic coupling. Physical Review X.

[11] Macke JH, Opper M, Bethge M (2011). Common input explains higher-order correlations and entropy in a simple model of neural population activity. Physical Review Letters.

[12] Heiligenthal S (2011). Strong and weak chaos in nonlinear networks with time-delayed couplings. Physical review letters.

[13] Jahnke S, Memmesheimer R-M, Timme M (2008). Stable irregular dynamics in complex neural networks. Physical Review Letters.

[14] Timme M, Wolf F, Geisel T (2003). Unstable attractors induce perpetual synchronization and desynchronization. Chaos: An Interdisciplinary Journal of Nonlinear Science.

[15] Opper M, Winther O (1996). Mean field approach to Bayes learning in feed-forward neural networks. Physical review letters.

[16] Biehl M, Schwarze H (1995). Learning by online gradient descent. Journal of Physics A.

[17] Watkin TL, Rau A, Biehl M (1993). The statistical mechanics of learning a rule. Reviews of Modern Physics.

[18] Kinouchi O, Caticha N (1992). Optimal generalization in perceptions. Journal of Physics A: mathematical and General.

[19] Opper M, Haussler D (1991). Generalization performance of Bayes optimal classification algorithm for learning a perceptron. Physical Review Letters.

[20] Kinzel W, Rujan P (1990). Improving a network generalization ability by selecting examples. EPL (Europhysics Letters).

[21] Legg S, Hutter M (2007). Universal intelligence: A definition of machine intelligence. Minds and Machines.

[22] Ostojic S (2014). Two types of asynchronous activity in networks of excitatory and inhibitory spiking neurons. Nature neuroscience.

[23] Akam T, Kullmann DM (2014). Oscillatory multiplexing of population codes for selective communication in the mammalian brain. Nature Reviews Neuroscience.

[24] Renart A (2010). The asynchronous state in cortical circuits. science.

[25] Marblestone AH, Wayne G, Kording KP (2016). Toward an integration of deep learning and neuroscience. Frontiers in computational neuroscience.

[26] Cassenaer S, Laurent G (2012). Conditional modulation of spike-timing-dependent plasticity for olfactory learning. Nature.

[27] Markram H, Gerstner W, Sjöström PJ (2012). Spike-timing-dependent plasticity: a comprehensive overview. Frontiers in synaptic neuroscience.

[28] Park Y, Choi W, Paik S-B (2017). Symmetry of learning rate in synaptic plasticity modulates formation of flexible and stable memories. Scientific Reports.

[29] Kragic D (2018). From active perception to deep learning. Science Robotics.

[30] Jo Y (2017). Holographic deep learning for rapid optical screening of anthrax spores. Science advances.

[31] Barra A, Bernacchia A, Santucci E, Contucci P (2012). On the equivalence of hopfield networks and boltzmann machines. Neural Networks.

[32] Sardi S (2018). Adaptive nodes enrich nonlinear cooperative learning beyond traditional adaptation by links. Scientific reports.

[33] Rosenblatt F (1958). The perceptron: a probabilistic model for information storage and organization in the brain. Psychological review.

[34] Kotsiantis SB, Zaharakis I, Pintelas P (2007). Supervised machine learning: A review of classification techniques. Emerging artificial intelligence applications in computer engineering.

[35] Brette R, Gerstner W (2005). Adaptive exponential integrate-and-fire model as an effective description of neuronal activity. Journal of neurophysiology.

[36] Spruston N (2008). Pyramidal neurons: dendritic structure and synaptic integration. Nature Reviews Neuroscience.

[37] Rosen-Zvi M, Klein E, Kanter I, Kinzel W (2002). Mutual learning in a tree parity machine and its application to cryptography. Physical Review E.

[38] Risken, H. In *The Fokker-Planck Equation* 63–95 (Springer, 1996).

[39] Ambrogio S (2018). Equivalent-accuracy accelerated neural-network training using analogue memory. Nature.

[40] Diederich S, Opper M (1987). Learning of correlated patterns in spin-glass networks by local learning rules. Physical review letters.

[41] Barra A, Beccaria M, Fachechi A (2018). A new mechanical approach to handle generalized Hopfield neural networks. Neural Networks.

[42] Agliari E (2015). Hierarchical neural networks perform both serial and parallel processing. Neural Networks.

[43] Spitzner P, Kinzel W (1989). Freezing transition in asymmetric random neural networks with deterministic dynamics. Zeitschrift für Physik B Condensed Matter.

