# Supplementary material for: Biological learning curves outperform existing ones in artificial intelligence algorithms
Source: Sci Rep. 2019 Aug 9;9:11558. doi: 10.1038/s41598-019-48016-4 (PMC6688986; doi:10.1038/s41598-019-48016-4)
Supplement: Supplementary file 1 — Supplementary Information [file 41598_2019_48016_MOESM1_ESM.pdf]

Supplementary figures

Biological learning curves outperform existing ones in artificial intelligence algorithms

Herut Uzan[1,†], Shira Sardi[1,†], Amir Goldental[1], Roni Vardi[1] & Ido Kanter[1,2,*]

[1]Department of Physics, Bar-Ilan University, Ramat-Gan, 52900, Israel.

[2]Gonda Interdisciplinary Brain Research Center and the Goodman Faculty of Life Sciences, Bar-Ilan University, Ramat-Gan, 52900, Israel.

[†]These authors contributed equally to this work.

[*]e-mail: ido.kanter@biu.ac.il

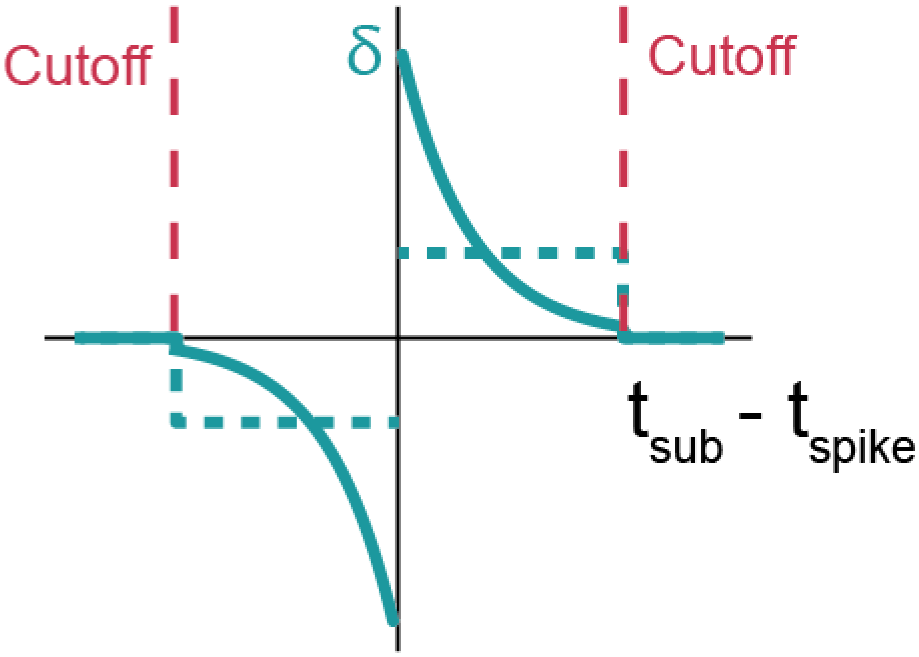

Figure S1. The adaptation rule. A typical STDP profile δ=A·exp(-|t|/15)·sign(t) (solid turquoise line), where t, measured in ms, indicates the time-lag between a sub-threshold stimulation, t_{sub}, arriving from one synapse/dendrite and another stimulation which generates a spike, t_{spike}, arriving from a different synapse/dendrite, respectively. A simplified two-level adaptation rule, δ=±A (dashed turquoise line). Both adaptive rules have a cutoff at 50 ms (dashed red lines).

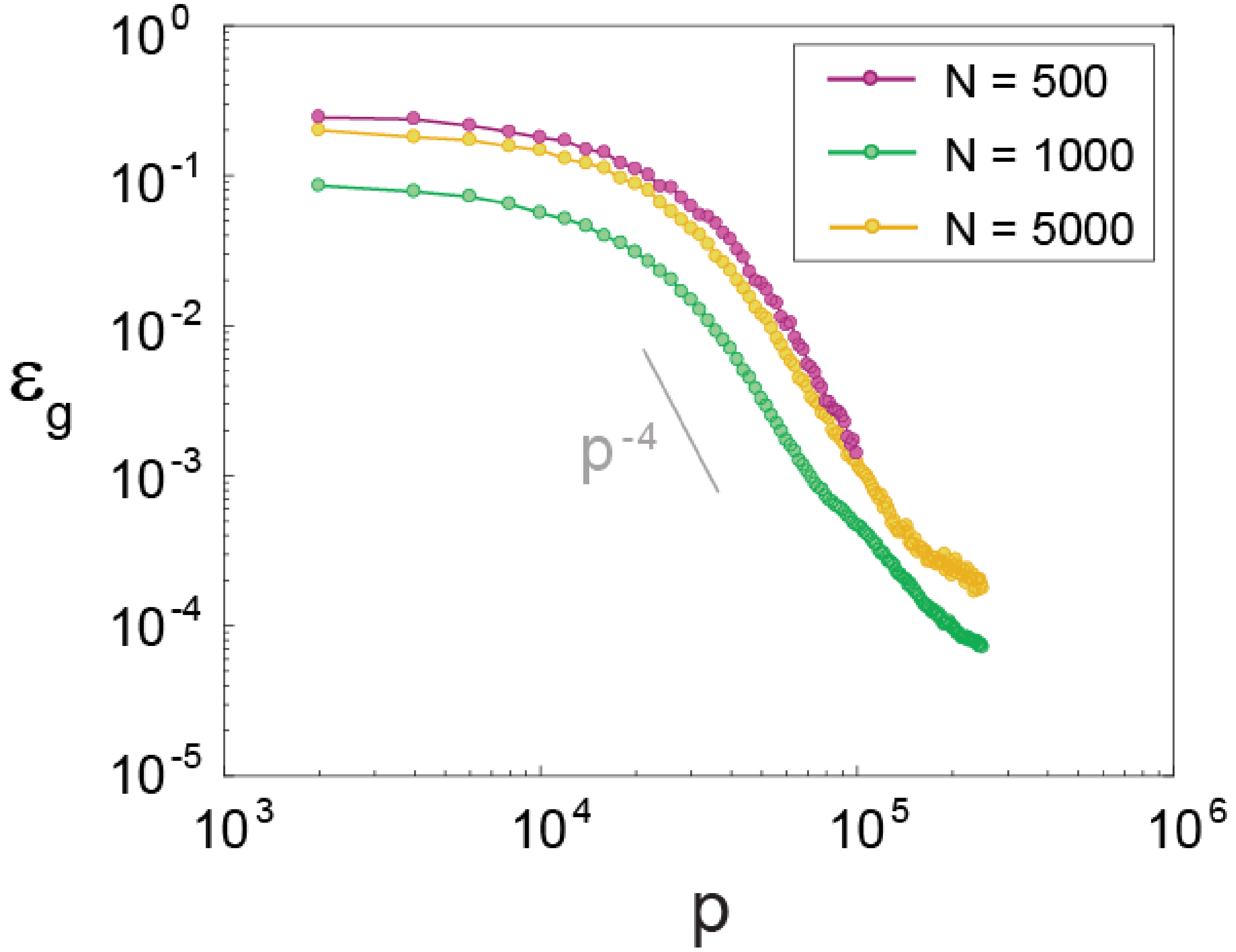

Figure S2. The student performs STDP following its own outputs. Generalization error, ε_g, as a function of the number of examples, p, for the same dynamics as in Fig. 2B, but the student performs adaptation steps (STDP) following its own outputs. Power-law slop of p is presented as a guideline.

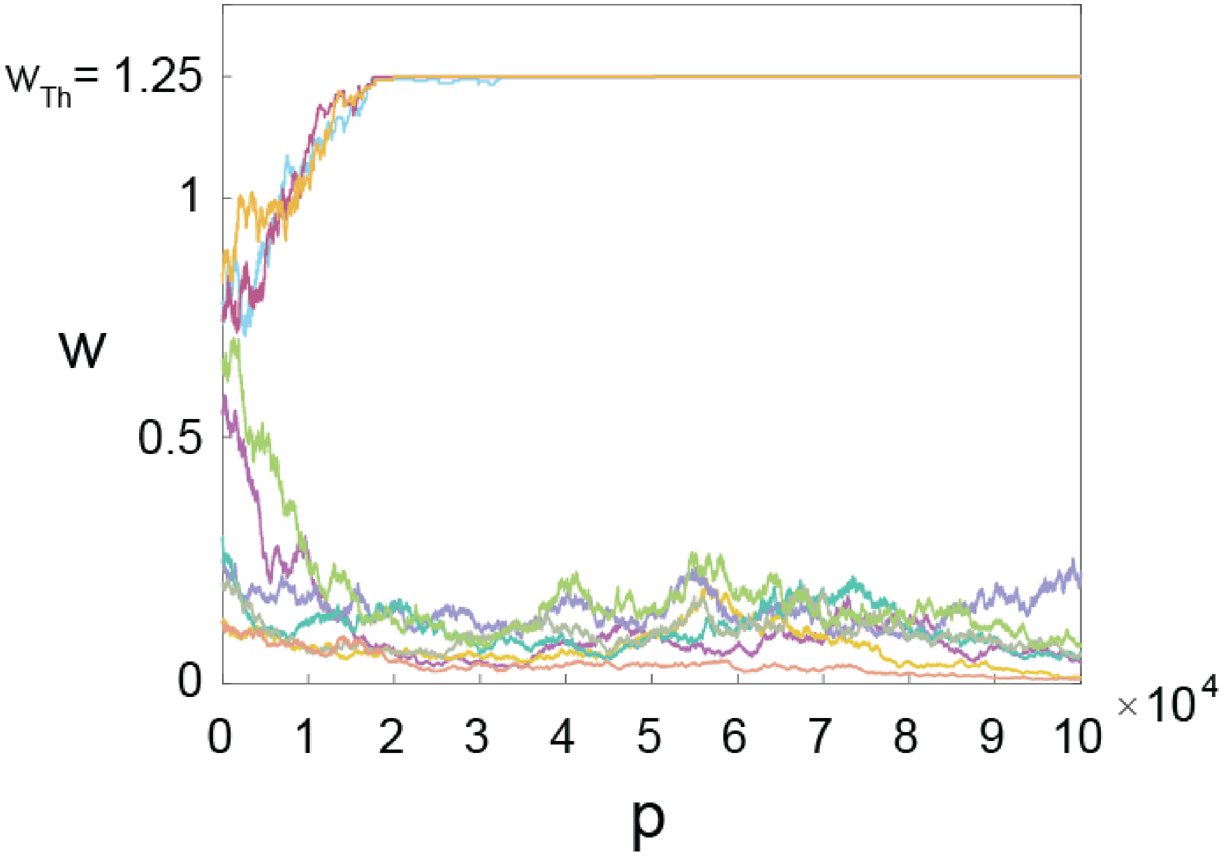

Figure S3. Dynamics of synaptic weight strengths. An example of the dynamics of ten weights taken from Fig. 2B with N=1000, indicating that weights dynamically converge towards extreme values.

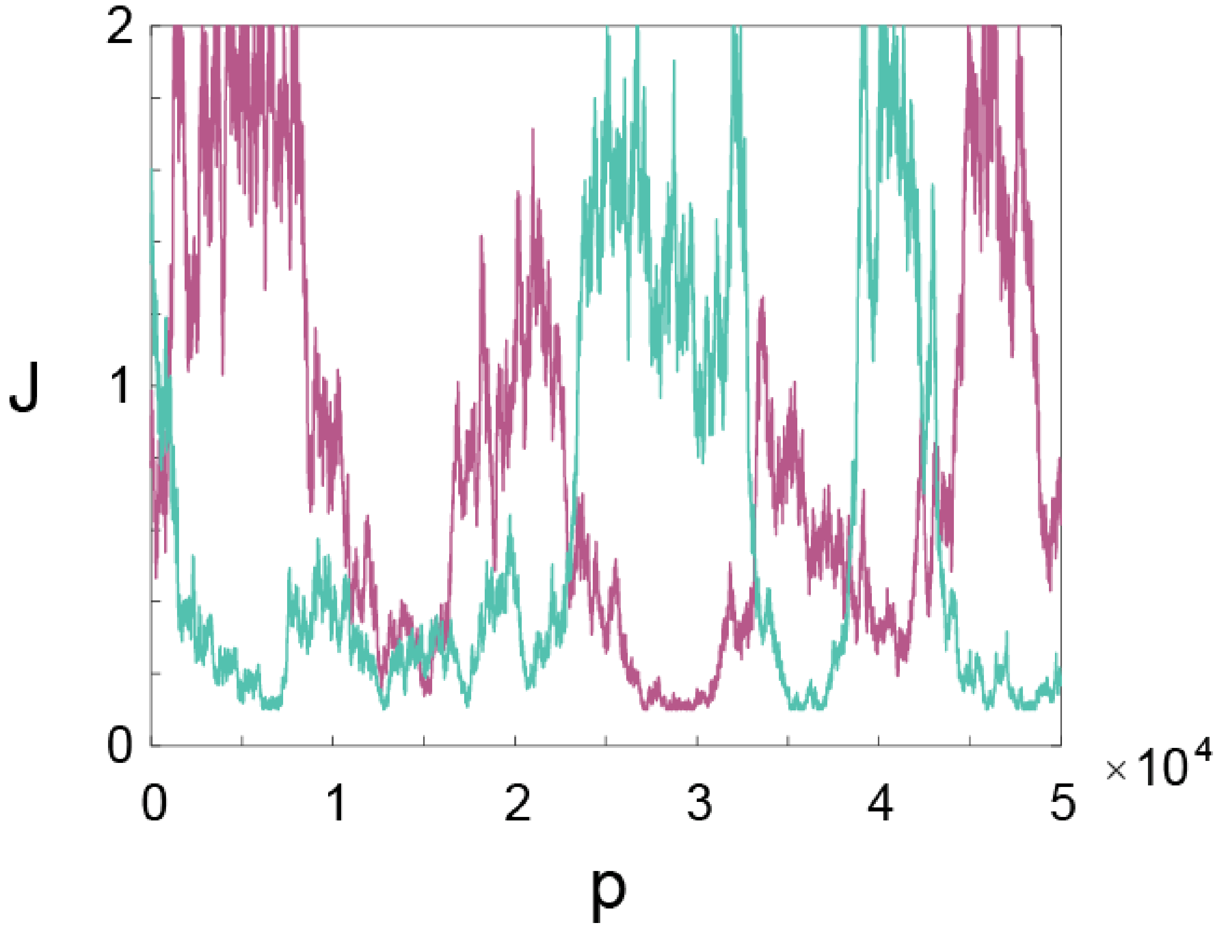

Figure S4. Oscillations of the dendritic strengths. Example of two dendritic strengths, J, taken from Fig. 3B with N=1000, indicating that the strengths, J, are constantly changing throughout the entire allowed range of values [0.1, 2].

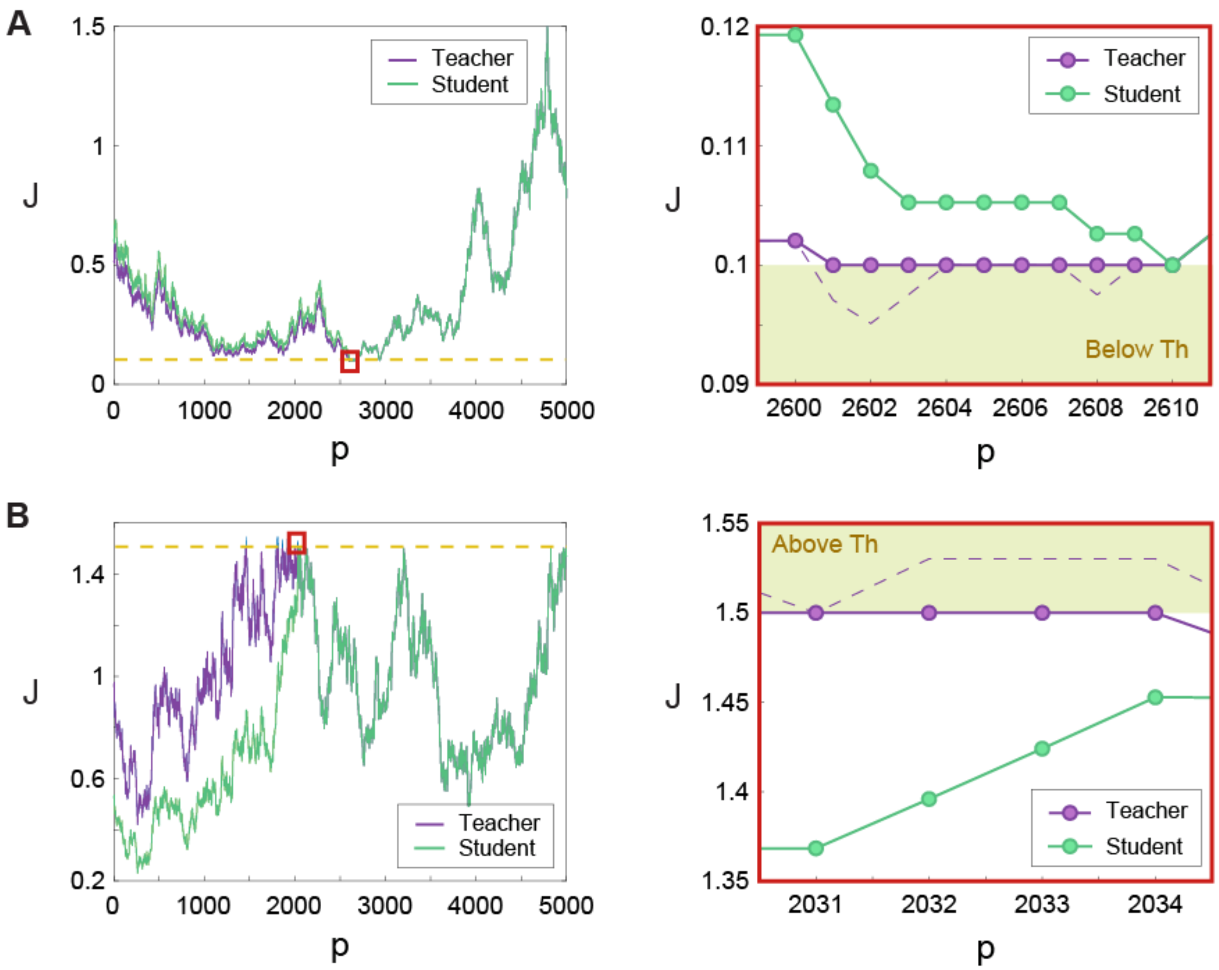

Figure S5. Hits at the boundary values as a mechanism for tracking the teacher oscillating dendritic strengths by the student. (**A**), Example of a dendritic strength of the teacher and the student (color coded). The strength of the student synchronizes with the strength of the teacher around the lower bound, J=0.1. A zoom-in of the red area is presented (right), where the dashed line represents the expected strengths of the teacher without considering the lower bound. Each hit results in a decreased gap between the strengths of the teacher and the student. (**B**), Similar to (**A**) but in the vicinity of the upper bound, J=1.5. The dynamics parameters were as in Fig. 3B with A = 0.005 and J_i was bounded from below by 0.1 and from above by 1.5.

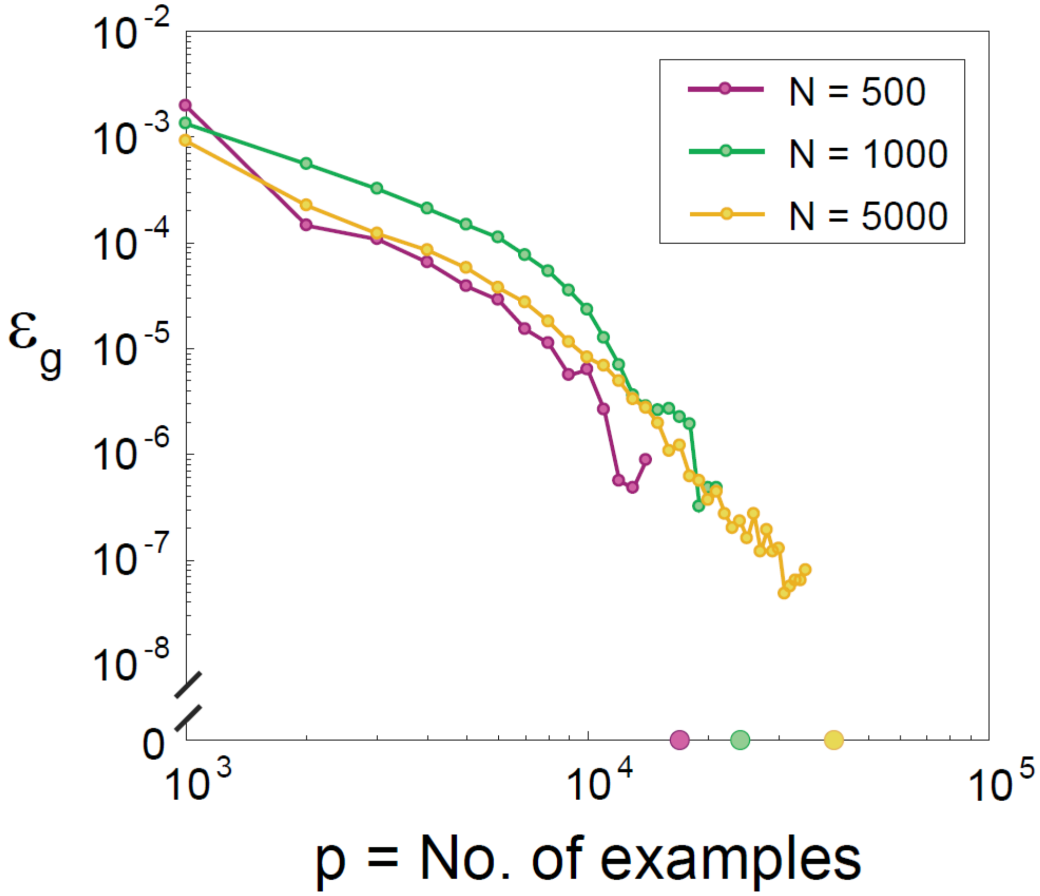

Figure S6. Dendritic learning with step size, λ, independent of N. The generalization error, ε_g, scales with the number of examples, p, for the same dynamics as in Fig. 3E, but with learning step size independent of N, λ=0.001 (equation 2).

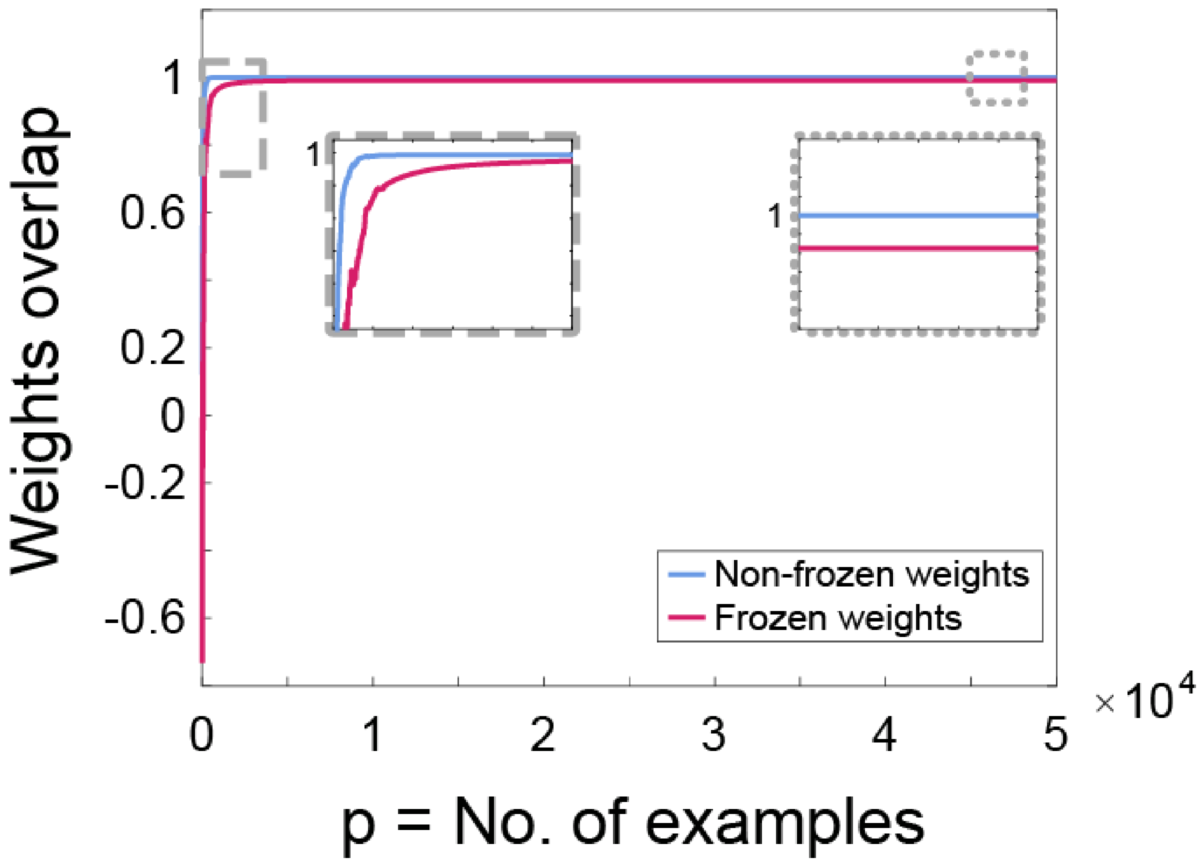

Figure S7. The overlap of frozen and non-frozen dendritic strengths. Overlap of the dendritic strengths between the teacher and the student for the dynamics presented in Fig. 3E. The overlap of the non-frozen dendritic strengths (blue line) and the overlap of the frozen dendritic strengths (red line). A zoom-in (dashed rectangle) indicates the fast convergence of the overlap of the non-frozen dendritic strengths to 1. A zoom-in (dotted rectangular) indicates the much slower convergence of the overlap of the frozen dendritic strengths to a constant below 1 (all frozen dendrites are above-threshold, but with different strengths).

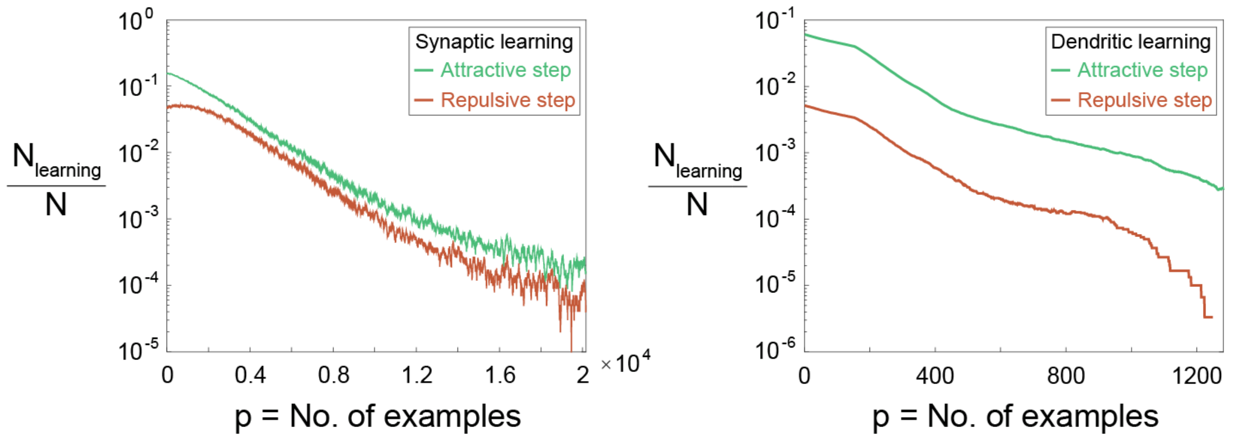

Figure S8. Statistics of attractive and repulsive learning steps. Left: The fraction of updated synaptic strengths as a function of p with attractive/repulsive (green/red) learning steps (Methods) with the same parameters as in Fig. 2B. Right: Similarly for dendritic learning, Fig. 3B. The data was smoothed using a sliding window of 100 examples.
